# Registration of catheter-related complications in adverse events reporting systems: a major underestimation of the real complication practice

**DOI:** 10.1177/17571774211012455

**Published:** 2021-06-22

**Authors:** Bart J Laan, Mieke H Godfried, Suzanne E Geerlings

**Affiliations:** 1 Amsterdam UMC, University of Amsterdam, Internal Medicine, Infectious Diseases, Amsterdam, The Netherlands; 2 Amsterdam UMC, University of Amsterdam, Internal Medicine, Amsterdam, The Netherlands

**Keywords:** Healthcare-associated infections, patient safety, safety management

## Abstract

Reporting and learning from preventable adverse events is crucial to improve patient safety. Although physicians should file and analyse adverse events by law in The Netherlands, it is unknown if these reporting systems are sufficiently used in clinical practice. This study is a substudy of the multicenter RICAT trial, a successful quality improvement project to reduce inappropriate use of intravenous and urinary catheters in medical wards in seven hospitals, in which we screened 5696 patients and documented 803 catheter-related complications. We also checked the adverse events reporting systems of these patients and found that only 13 (1.6%) of 803 catheter-related complications were registered. Of the infectious complications only five (10.9%) of 46 catheter-associated bloodstream infections and urinary tract infections were registered. We conclude that the reported complications were a major underestimation of the real complication practice in medical wards in The Netherlands.

The RICAT trial is registered at Netherlands Trial Register, trial NL5438.

## Background

‘First, do no harm’ is one of the most familiar oaths for healthcare professionals. To improve patient safety it is crucial to report and learn from preventable adverse events. Adverse events are unintended and unfavourable injuries caused by healthcare management, which result in the need for additional treatment or permanent harm (Gallagher et al, 2006). Although the Institute of Medicine has already published an alarming landmark report, *To Err Is Human*, to cause awareness about medical errors in 1999, a systematic review in 2008 still showed that in-hospital adverse events are a serious problem, with a prevalence of 9%, of which 7% were lethal (de Vries et al, 2008). A substantial proportion (44%) of adverse events was regarded as preventable (de Vries et al, 2008).

To reduce preventable adverse events, it is essential to keep track of them to analyse trends and start improvement strategies. Therefore, voluntary reporting systems for adverse events and near misses are compulsory for accreditation of the joint commission, and some countries have introduced national patient safety surveillance systems. In The Netherlands, a physician should inform the patient about an adverse event and file the event in the electronic patient record (Healthcare Quality, Complaints and Disputes Act (WKKGZ), article 10:3). Further, the physician will register the adverse event in a reporting system, and the department should structurally analyse risk factors and clinical results of adverse events.

In a survey in three teaching hospitals in the United States, only 55% of physicians and residents knew how to report adverse events, and only 40% knew what kind of adverse events had to be reported (Kaldjian et al, 2008). Although physicians should file and analyse adverse events in The Netherlands, it is unknown if these reporting systems are sufficiently used in clinical practice. As healthcare-associated infections, such as catheter-associated bloodstream infections and urinary tract infections, are one of the most common types of adverse events (Schwendimann et al, 2018), we evaluated the registration of catheter-related infections in adverse event reporting systems and compared the results with our own measurement in medical records. We hypothesised that 25% of the adverse events would be registered in the reporting systems.

## Methods

We performed a substudy of our multicentre, interrupted time series, entitled the RICAT study (Reduce Inappropriate use of intravenous and urinary CATheters), to reduce the inappropriate use of intravenous and urinary catheters in the internal medicine and non-surgical subspecialty wards in seven hospitals (three university and four general hospitals) in The Netherlands from 1 September 2016 to 1 April 2018 (Laan et al, 2020). We prospectively included adult patients who had a (central and/or peripheral) venous and/or urinary catheter. Further details are described in the original study publication (Laan et al, 2020).

Ethical approval was obtained from the Medical Ethics Research Committee of the Academic Medical Centre, with a waiver for individual informed consent. Local feasibility was approved by the local institutional review boards of the participating hospitals. The results are reported in accordance with the Strengthening the Reporting of Observational Studies in Epidemiology (STROBE) statement.

To verify the current use of adverse event reporting systems, we additionally performed a chart review 30 days after discharge. We collected information on whether patients had a catheter-related complication during hospital stay, and if these complications were registered as part of regular care in adverse event reporting systems. Three hospitals used EPIC Systems Corporation, two hospitals used HiX by ChipSoft, and one hospital used SAP software for electronic health records. In EPIC the adverse events reporting system is integrated in the patient records.

### Study outcomes

The primary outcome was the percentage of catheter-related infections (defined as central line, and peripheral venous catheter-associated bloodstream infections, and catheter-associated urinary tract infections) registered in the adverse event reporting system. As the systems used no formal definitions of catheter-related infections, we used clinician-based definitions of catheter-related infections. Secondary outcomes were other registered catheter-related complications, such as extravasation, haematoma, thrombosis and decompensation by fluid overload for venous catheters, and haematuria and urethral trauma for urinary catheters. Adverse events were defined as catheter-related complications, as described above, that needed additional treatment. If a patient had more than one complication, we only reported one complication per catheter.

### Statistical analysis

We summarised categorical data as frequency and percentage. We did a subgroup analysis for the hospitals with an integrated adverse events reporting system in the electronic patients records. Descriptive analyses were performed using IBM SPSS Statistics (version 25.0). The RICAT study is registered at Netherlands Trial Register, trial NL5438.

## Results

Between 1 September 2016 and 1 April 2018, we screened for the RICAT study 5696 patients by direct patients observations, 3577 (62.8%) had a peripheral venous catheter, 722 (12.7%) a urinary catheter and 191 (3.4%) a central venous catheter. In total, 803 catheter-related complications were found in patient records (Table 1). Data on the complications were not available in one university hospital.

**Table 1.** Registration of catheter-related complications.

Complications	Data: health records (measured in RICAT study)^a^	Data: reporting system^a^	Total measured complications from health records registered in reporting system
Central venous catheters	30/171 (17.5)	1/171 (0.6)	1/30 (3.3)
CLA-BSI	6/171 (3.5)	0/171 (0.0)	0/6 (0.0)
Peripheral venous catheters	682/3287 (20.7)	7/3287 (0.2)	7/682 (1.0)
PVC-BSI	6/3287 (0.2)	1/3287 (0.0)	1/6 (16.7)
Phlebitis	328/3287 (10.0)	6/3287 (0.2)	6/328 (1.8)
Urinary catheter	91/675 (13.5)	5/675 (0.7)	5/91 (5.5)
CA-UTI	34/675 (5.0)	4/675 (0.6)	4/34 (11.8)
Total	803/4133 (19.4)	13/4133 (0.3)	13/803 (1.6)

Data are *n*/*N* (%).

Data from six hospitals.

CLA-BSI: central line-associated bloodstream infection; PVC-BSI: peripheral venous catheter-associated bloodstream infection; CA-UTI: catheter-associated urinary tract infection.

a Denominators are numbers of patients having the specific catheter.

We found that 13 (1.6%) of all 803 catheter-related complications, and five (10.9%) of 46 catheter-related infections were registered in the adverse event reporting system. Details are provided in Table 1. One peripheral venous catheter-associated bloodstream infection, six phlebitis, four catheter-associated urinary tract infections, one case of haematuria and one case of fluid overload due to a central venous catheter were registered as a complication. The sensitivity of the adverse event reporting system for catheter-related infections was 0.02.

Sensitivity analysis in the hospitals with adverse event reporting systems integrated in patient records showed only modest improvement of registrations. In total, 12 (2.8%) of 422 catheter-related complications were registered compared to 13 (1.6%) of 803 complications. We found no differences for specific types of catheters or for catheter-related infections.

## Discussion

We showed that the registration of catheter-related infections and other complications in adverse event reporting systems is highly underused in six hospitals in The Netherlands. It was even worse than our hypothesis of 25%. This leads to an underestimation of the prevalence of (preventable) catheter-related infections, and as a result a lack of learning opportunity to recognise, investigate and reduce preventable adverse events.

A survey in Australia showed that most healthcare workers were aware of the adverse event reporting system (Evans et al, 2006). However, only a small percentage of doctors report adverse events. Barriers to adverse event reporting appear to be multifactorial, such as not knowing how and what to report, long forms, lack of time and inadequate feedback regarding previous reports (Evans et al, 2006).

Our findings are similar to a retrospective review in a large hospital in England where a routine incident reporting system identified only 5% of adverse events (Sari et al, 2007). In contrast, the nationwide routine reporting of surgical adverse events in The Netherlands is integrated in daily clinical practice, which results in a registration of 85% of serious adverse events (Marang-van de Mheen et al, 2005).

The main limitation of this study is that we specifically evaluated the registration of catheter-related events instead of all adverse events. Therefore, the outcome might not be generalisable for all other adverse events. However, healthcare-associated infections, mostly associated with the use of a catheter, are identified as an important patient safety challenge, because a substantial amount of catheter-related infections are preventable. Next, we did not evaluate the sentinel events, which are mandatorily reported to the Dutch Health Inspectorate and therefore could have a good registration rate.

Human errors that lead to adverse events cannot be eliminated. However, we could better identify the problem and implement adverse event reporting systems that recognise and result in actions to prevent adverse events. This could be through quality improvement projects that focus on the involvement of residents, because they are at the frontline of patient care and the medical specialists of the future. A successful example is an educational intervention in anaesthesiology residents in Chicago, which led to improved adverse event reporting by residents (0 per quarter to almost 30 per quarter) and learning opportunities that resulted in process improvements of anaesthesia care (Jericho et al, 2010). However, it remains challenging to use administrative data or adverse reporting systems for surveillance, because it is resource intensive and lacks standardisation. Methods and indicators used in surveillance systems are often lacking (Núñez-Núñez et al, 2018). The development of automated surveillance systems could address these challenges (van Mourik et al, 2018). Next to good registration, it is crucial to analyse serious adverse events structurally; for example, in a root cause analysis, to identify underlying factors that increase the likelihood of adverse events and introduce improvement projects to tackle these underlying factors. So, reporting systems should be an opportunity to learn from adverse events and improve patient safety through an appropriate safety culture (Mitchell et al, 2016).

As health care is a very complex system, human errors are inevitable, but systems must be developed to minimise preventable adverse events. The first step is to know how many and what kind of preventable adverse events occur on each specific ward or department by registration. However, our results show that this crucial action is lacking and is highly underused in adverse event reporting systems from six hospitals in The Netherlands. Improvement projects should start to increase the registration of adverse events. To improve patient safety, reporting systems should detect most adverse events, identify the underlying problem, develop interventions and monitor improvements to reduce adverse events sustainably.

## Supplemental Material

sj-docx-1-bji-10.1177_17571774211012455 – Supplemental material for Registration of catheter-related complications in adverse events reporting systems: a major underestimation of the real complication practice Click here for additional data file. Supplemental material, sj-docx-1-bji-10.1177_17571774211012455 for Registration of catheter-related complications in adverse events reporting systems: a major underestimation of the real complication practice by Bart J Laan, Mieke H Godfried and Suzanne E Geerlings in Journal of Infection Prevention
