# Supplementary material for: Registration of catheter-related complications in adverse events reporting systems: a major underestimation of the real complication practice
Source: J Infect Prev. 2021 Jun 22;23(1):11–4. doi: 10.1177/17571774211012455 (PMC8811235; doi:10.1177/17571774211012455)
Supplement: sj-docx-1-bji-10.1177_17571774211012455 – Supplemental material for Registration of catheter-related complications in adverse events reporting systems: a major underestimation of the real complication practice [file sj-docx-1-bji-10.1177_17571774211012455.docx]

**Supplementary Appendix**

**Definitions of catheter-related infections**

For the RICAT-study, catheter-related infections were defined according to the National Healthcare Safety Network criteria (see below).^1^ However, the reporting systems in clinical practise do not use a formal definitions of catheter-related infections. Therefore for this study, we used clinician-based definitions of catheter-related infections, defined as a catheter-related complication, that required additional treatment, according the medical record.

For catheter-associated bloodstream infection a patient must meet all four criteria below:

1. Catheter was in place for >2 days on the date of event, with day of placement being day 1, and was in place on the date of event or the day before.

2. Patient has at least one of the following signs or symptoms: fever (>38·0°C), chills, or hypotension.

3. Organism(s) identified from blood is not related to an infection at another site.

4. The same common commensal is identified from two or more blood specimens drawn on separate occasions.

For catheter-associated urinary tract infection a patient must meet all three criteria below:

1. Urinary catheter was in place for >2 days on the date of event, with day of placement being day 1, and was in place on the date of event or the day before.

2. Patient has at least one of the following signs or symptoms: fever (>38·0°C), suprapubic tenderness, costovertebral angle pain or tenderness, urinary urgency, urinary frequency, or dysuria.

3. Patient has a urine culture with no more than two species of organisms identified, at least one of which is a bacterium of ≥10^5 CFU/ml.

For phlebitis a patients must have two or more of the following symptoms: local pain, warmth, tenderness, erythema, or a palpable cord along the vein.

**References**

1. CDC. National Healthcare Safety Network (NHSN) Overview 2016. http://www.cdc.gov/ (accessed May 26, 2016).
